# First-principles study on the structural and electronic properties of metallic HfH_2_ under pressure

**DOI:** 10.1038/srep11381

**Published:** 2015-06-22

**Authors:** Yunxian Liu, Xiaoli Huang, Defang Duan, Fubo Tian, Hanyu Liu, Da Li, Zhonglong Zhao, Xiaojing Sha, Hongyu Yu, Huadi Zhang, Bingbing Liu, Tian Cui

**Affiliations:** 1 State Key Laboratory of Superhard Materials, College of Physics, Jilin University, Changchun 130012, People’s Republic of China; 2 Department of Physics and Engineering Physics, University of Saskatchewan, Saskatoon, Canada, S7N 5E2

## Abstract

The crystal structures and properties of hafnium hydride under pressure are explored using the first-principles calculations based on density function theory. The material undergoes pressure-induced structural phase transition *I*4/*mmm*→*Cmma*→*P*2_1_/*m* at 180 and 250 GPa, respectively, and all of these structures are metallic. The superconducting critical temperature *T*_c_ values of *I*4/*mmm*, *Cmma*, and *P*2_1_/*m* are 47–193 mK, 5.99–8.16 K and 10.62–12.8 K at 1 atm, 180 and 260 GPa, respectively. Furthermore, the bonding nature of HfH_2_ is investigated with the help of the electron localization function, the difference charge density and Bader charge analyses, which show that HfH_2_ is classified as a ionic crystal with the charges transferring from Hf atom to H.

Up to now, hydrogen, the simplest and most abundant element of universe is still fascinating to physics community. The studies about hydrogen are mainly reflected in two aspects. One is the metallic and superconducting of hydrogen. The other is considered as the most promising clean energy sources with the capability of replacing fossil fuels. The former is going back to the early 1930: hydrogen was predicted to be a potential high *T*_*c*_ superconductor at high pressures^1^,^2^,^3^, because of its low mass density and high elastic stiffness. However, hydrogen remains an insulator, despite considerable ongoing experimental effort up to pressure of 300 GPa^4^. Recently, hydrogen dense materials has been as a potential route to achieve metallization with high *T*_*c*_ at lower pressure. For example, high *T*_*c*_ with 190 K in the sulfur hydrides at 200 GPa has been found by both of theoretical predicted and experimental observation^5^,^6^,^7^. The latter is still limited by many practical and technological factors, which requires the development of safe and efficient hydrogen storage technology.

Hydrogen can also react with transition metal elements formed metal hydrides under certain conditions and they can keep stable under ambient conditions, such as YH_2_, YH_3_, TiH_2_, ZrH_2_, HfH_2_, etc^8^,^9^,^10^,^11^. In addition, for transition metal, the number of d-shell electrons per atom is import for the *T*_*c*_, which is correlate with the important parameters of the Bardeen–Cooper–Schrieffer theory about superconductivity^12^. Transition metal hydrides not only are regarded as promising potential materials for storing hydrogen^13^, but also exhibit fascinating superconducting properties. For example, the superconducting transition temperature *T*_*c*_ of Th_4_H_15_ is 8 K at ambient conditions^14^. Many transition-metal dihydrides TMH_2_ can form CaF_2_ (*Fm*–3*m* space group) crystal structure. And in this structure, the metal atoms form a face-centered-cubic sublattice while the hydrogen atoms occupy the tetrahedral lattice sites. However, the group IVB dihydrides TiH_2_, ZrH_2_, and HfH_2_ show a basically face-centered-tetragonal (fct) cell structure (*I*4/*mmm* space group). And these dihydrides (TiH_2_, ZrH_2_, and HfH_2_) are applied in various fields. For instances, TiH_2_ can be served as the catalyst in polymerization reaction, which is interesting in the new quenchable phases^15^,^16^. ZrH_2_ is widely used as a neutron moderator in nuclear reactor^17^,^18^,^19^. Hafnium hydride, instead of boron carbide, is a perfect neutron control materials for fast reactors^20^.

The high-pressure development has become a vibrant area of research. Not only does pressure provide a different route to the synthesis of new compounds, but also enables many known materials to exhibit novel phenomena that cannot be found at normal conditions. Lately, theoretical study revealed that TiH_2_ at high pressure had a structural transformations *I*4/*mmm*→*P*4/*nmm*→*P*2_1_/*m* and the calculated pressure of phase transition were 63 and 294 GPa, respectively^21^. The structures and properties of ZrH_2_ were also investigated in experiment under high pressure^22^. Despite large amounts of theoretical and experimental researches on HfH_2_^23^, there is relatively little investigation on its new structures, chemical bonding nature, dynamical properties and superconductivity under high pressures. Therefore, great attentions are needed to explore the high–pressure structures of HfH_2_.

In this paper, we examine in detail the optimum static structures of HfH_2_ system at zero temperature by using the newly developed *ab initio* evolutionary algorithm. Moreover, we employ a first-principles method to calculate their dynamical stability and electronic band structures. Our calculated results show that HfH_2_ adopts the *I*4*/mmm* structure at low pressures. On compression, *Cmma* phase possesses the lowest enthalpy, then at higher pressures *P*2_1_/*m* becomes energetically favorable. Band structures and density of states indicate that these structures are metallic. The estimated *T*_*c*_ are 47–193 mK at 1 atm, 5.99–8.16 K at 180 GPa and 10.62–12.8 K at 260 GPa, for *I*4/*mmm*, *Cmma* and *P*2_1_/*m*, respectively. The electron localization function (ELF), the difference charge density and Bader charge analysis show that HfH_2_ is an ionic crystal with the charge transferring from Hf to H atom. Our present study attempts to provide a better understanding of the pressure-induced phase transformations and properties of HfH_2_ under pressure.

## Results and Discussion

**Figure 1:**
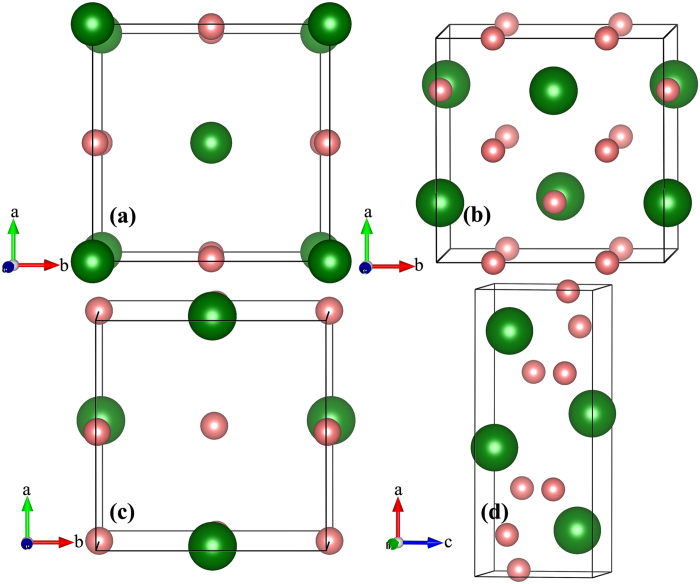
Crystal Structures of HfH_2_. The selected stable phases for HfH_2_. Green atoms depict Hf, while pink atoms present H, (**a**) *I*4/*mmm* at 1 atm, (**b**) *Cmma* at 200 GPa, (**c**) *P*4/*nmm* at 200 GPa and (**d**) *P*2_1_/*m* at 300 GPa.

The crystal structure prediction were performed with simulation sizes ranging from one to four HfH_2_ formula units (f.u.) at 1 atm, 50, 80, 100, 150, 200, 250 and 300 GPa. Analysis of the predicted structures gave us a shortlist of candidate structures with space groups *Fm*–3*m*, *I*4/*mmm*, *P*4*/nmm*, *Cmma*, *P*2_1_/*m* and *C*2/*m*. Figure 1 shows the structural motifs of HfH_2_ under high pressure. At 1 atm, we obtain two structures: a CaF_2_ type structure with space group *Fm*–3*m* (4 f.u./cell) and a centered tetragonal structure with space group *I*4/*mmm* (2 f.u./cell) (Fig. 1(a)). At ambient conditions, many transition-metal dihydrides exist with fcc structure (i.e., a CaF_2_ structure), where metal atoms form a face-centered cubic lattice, and the centers of tetrahedrons are occupied by H atoms. Note that in *I*4/*mmm* structure, Hf atoms form a body-centered tetragonal (bct) sublattice with its coordination number is 8, while the H atoms are located on the planes and present a one dimensional chain. Then we observed the most stable structure which possess simple tetragonal *P*4/*nmm* (4 f.u./cell) orthogonal *Cmma* (4 f.u./cell) at 200 GPa. In the above two phases, the metal atoms form a bct and fcc cell for *P*4/*nmm* and *Cmma*, respectively. For *Cmma*, the coordination number of Hf becomes 9. Finally, at 300 GPa, the monoclinic *P*2_1_/*m* phase (4 f.u. /cell) becomes the preferred one (Fig. 1(d)). And in the *P*2_1_/*m* structure, Hf atoms can form a little distorted bcc lattice and its coordination is 12. Within *P*2_1_/*m* structure, Hf site is not equivalent, occupying the crystallographic 2*e* site, while H atoms take four different 2*e* sites. The lattice parameters and atomic positions of selected structures at favored pressures are summarized in Table SI. In comparison, we observed that in both HfH_2_ and TiH_2_^21^, the locations of metal and hydrogen atoms are similar for *I*4/*mmm*, *P*4*/nmm* and *P*2_1_*/m*, respectively. In all above structures, the nearest H-H distances are 2.149 Å at 1 atm, much longer than that in the pure H_2_. Furthermore, with increasing pressure, H-H distances decrease and become 1.548 Å at 300 GPa, which indicate that there isn’t any bonding trend among H atoms.

**Figure 2:**
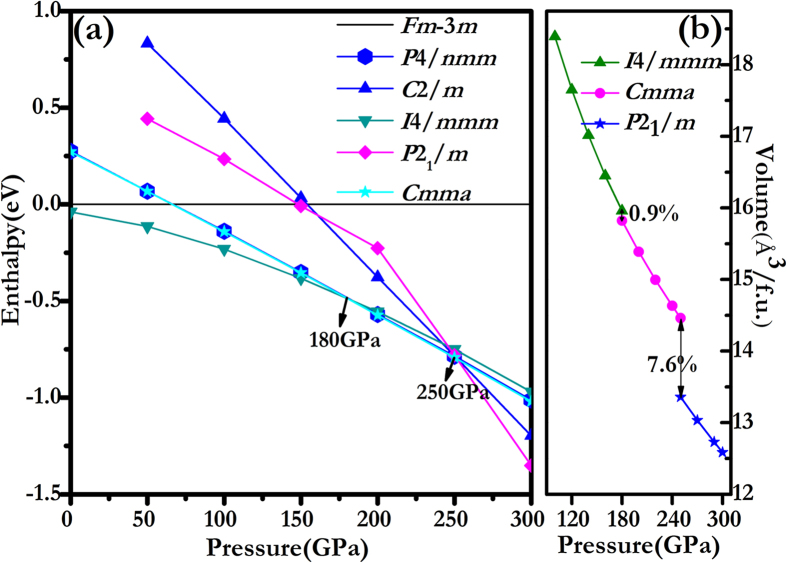
Enthalpy difference curves of HfH_2_ and EOS. (**a**) Calculated enthalpies per HfH_2_ unit of various structures relative to our predicted *Fm*–3*m* phase as a function of pressure range from 0 to 300 GPa. (**b**) Volume plotted as a function of pressure for the *I*4/*mmm*, *Cmma* and *P*2_1_/*m* structures.

The phase stability of HfH_2_ under pressure has been investigated systematically. Enthalpy differences as a function of pressure for these competitive solid structures together relative to *Fm*–3*m* in the pressure range from 0 to 300 GPa are plotted in Fig. 2(a). It is clearly seen that *I*4/*mmm* phase has lower enthalpies than all other candidates, which indicate it is the thermodynamic ground state. Above 180 GPa, *Cmma* (*P*4*/nmm*) becomes the most stable structure and remains up to 250 GPa. Then under higher pressures, a monoclinic *P*2_1_*/m* becomes more favored. In these two transitions, the coordination number of Hf increased from 8 to 9, 9 to 12, respectively. Surprisingly, we found that *Cmma* and *P*4*/nmm* are energetically nearly degenerated in the range of 0–300 GPa, and enthalpy difference is less than 1 meV/f.u.. If *I*4/*mmm*, *P*4*/nmm* and *P*2_1_*/m* phases satisfied the stability conditions of mechanics and dynamics, the phase transition sequence of HfH_2_ is similar to the TiH_2_^21^. However, we found that the *P*4*/nmm* is not stable both in dynamical and mechanical properties (see the later discussions). The calculated equations of state (EOS) depicted in Fig. 2(b) shows that the *I*4/*mmm*→*Cmma* and *Cmma*→*P*2_1_/*m* phase transitions are discontinuous changes in volume at the transition point with volume collapses of 0.9% and 7.6%, suggesting the two phase transitions are the first-order nature.

**Figure 3:**
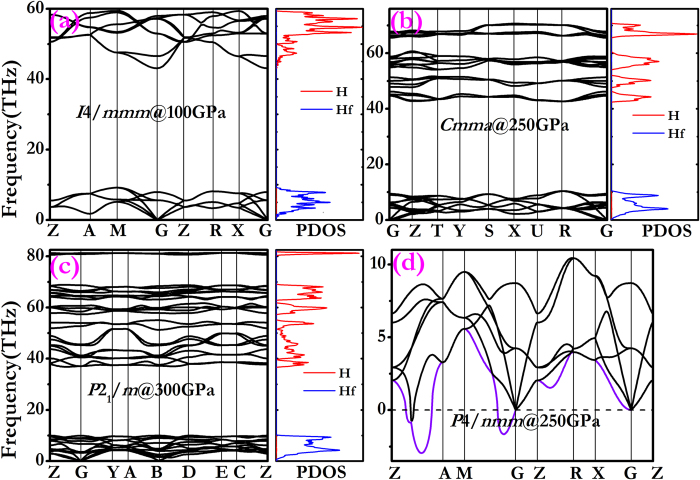
The phonon band structure and projected phonon DOS charts. (**a**–**d**) The phonon band structure and projected phonon DOS charts for *I*4/*mmm*, *P*4/*nmm, Cmma* and *P*2_1_/*m* at different pressure.

It is essential to determine the dynamical stability of the studied structures. The calculated phonon dispersion curves and projected phonon density of states (PHDOS) of the *I*4/*mmm*, *Cmma*, *P*4*/nmm* and *P*2_1_/*m* phases in the studied pressure range are presented in Fig. 3. The absence of any imaginary phonon frequencies in the entire Brillouin zone (Fig. 3(a–c)) confirms that *I*4/*mmm*, *Cmma* and *P*2_1_/*m* structures are dynamically stable regardless of the applied pressure. By contrast, there are imaginary phonon frequencies for *P*4*/nmm* phase (Fig. 3(d)) which indicates this phase is dynamically unstable. In addition, it is shown that two separate regions of phonon bands are clearly recognized. Since hafnium is much heavier than hydrogen atom, the vibration frequency of hafnium atom is obviously lower than that of hydrogen atom. And the low-frequency bands are merely from the Hf atoms, while higher-frequency modes are solely due to the light H atoms.

Mechanical property of the crystalline structure is one of the basic requirement when considering the phase stability. The elastic constants of *I*4/*mmm*, *Cmma*, *P*2_1_/*m* and *P*4/*nmm* structures were calculated at different pressures, as shown in Table SII. According to the mechanical stability criteria, the strain energy should be positive, which means the whole set of elastic constants matrix C_*ij*_ meet the Born-Huang stability criteria^24^. Obviously, the elastic constants of *I*4/*mmm*, *Cmma* and *P*2_1_/*m* meet the mechanical stability criteria, suggesting that the three structures are mechanically stable in our studied pressure range. However, elastic constants of *P*4*/nmm* phase cannot satisfy the stability conditions of mechanics for tetragonal crystal system owing to C_66_ < 0, which means it is mechanically unstable.

**Figure 4:**
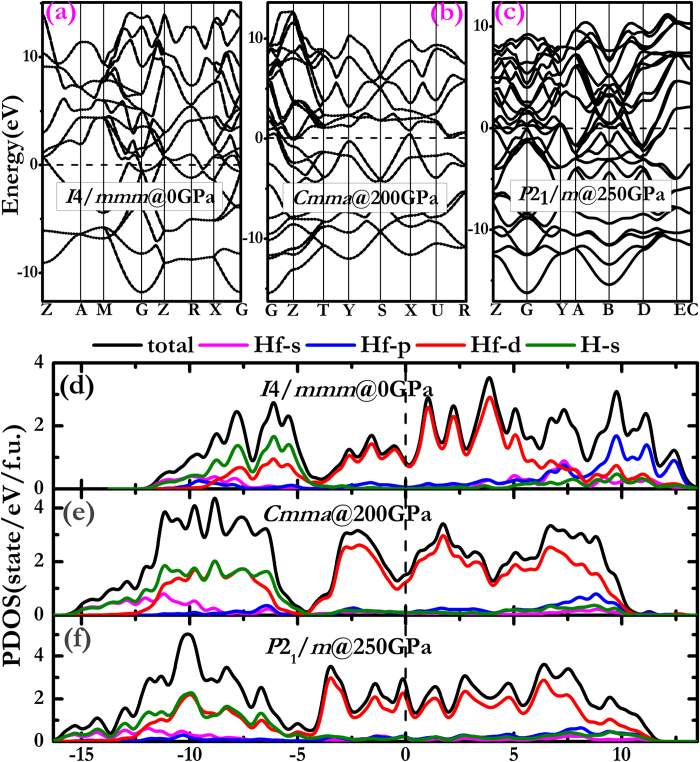
Electronic band structure and partial density of states (PDOS). (**a**–**f**) The calculated electronic band structure and PDOS for *I*4/*mmm* (0 GPa), *Cmma* (200 GPa) and *P*2_1_/*m* (250 GPa).

The electronic properties were studied by calculations of the electronic band structure and partial densities of states (PDOS) for *I*4/*mmm*, *Cmma* and *P*2_1_/*m* phases at different pressures, as presented in Fig. 4. Clearly, the overlap between the conduction and the valence bands for the three structures suggests that the above phases are metallic. From the PDOS of HfH_2_ (Fig. 4(d–f)), we see that the occupation properties of *I*4/*mmm*, *Cmma* and *P*2_1_/*m* phases are similar. And the pseudogap which is below the Fermi level, located at around −4.2 eV. The predominant feature of hybridization for H 1 s orbital and Hf 5d orbital is observed in the energy region below the pseudogap, while from the pseudogap to the Fermi level, the domination is Hf 5d states in the energy range. The metallic behavior of HfH_2_ indicates that this material might be a superconductor and we discussed it in the following.

**Figure 5:**
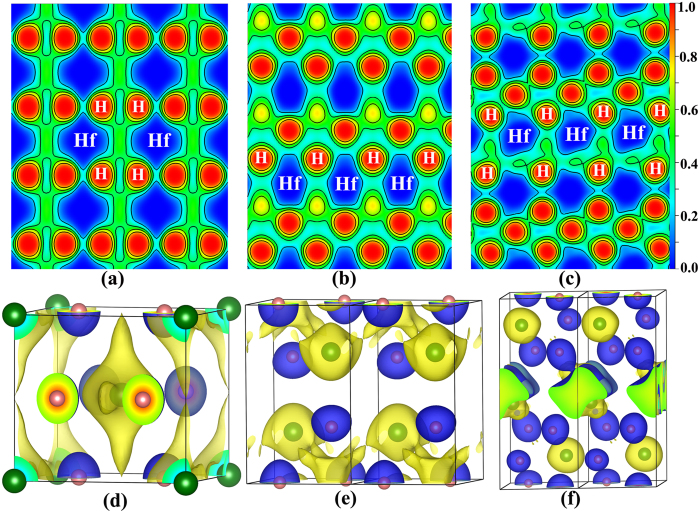
The electron localization function and difference charge density maps. (**a**–**c**) Electron localization function (ELF) maps of *I*4/*mmm* (100 GPa), *Cmma* (200 GPa) and *P*2_1_/*m* (250 GPa), respectively. (**d**–**f**) Difference charge density (crystal density minus superposition of isolated atomic densities) of *I*4/*mmm*, *Cmma* and *P*2_1_/*m* for HfH_2_ plotted at 100, 200 and 250 GPa, respectively. The isosurface value is set as: Blue represent positive (+0.05) while Yellow represent negative (−0.015).

In order to explore the bonding and analyze the ionic or covalent character of HfH_2_, the electron localization function (ELF) of *I*4/*mmm*, *Cmma* and *P*2_1_/*m* at 100 GPa, 200 GPa and 250 GPa, are plotted in Fig. 5(a–c). The maximum ELF value between Hf and H is less than 0.3, which suggests no covalent bonding. In order to further clearly understand the bonding nature, we have calculated the difference charge density (crystal density minus superposition of isolated atomic densities) of *I*4/*mmm* (100 GPa), *Cmma* (200 GPa) and *P*2_1_/*m* (250 GPa), as shown in Fig. 5(d–f). We can see charges transfer from Hf atom to H atom. The profile of the Hf is identical for *I*4/*mmm* and *Cmma* phases in Fig. 5(d,e), respectively. By contrast, *P*2_1_/*m* phase in Fig. 5(f) has two Hf’s contours owing to Hf sites are not equivalent, which occupy the crystallographic 2*e* site (Fig. 1(d)). To gain a better understanding of the bonding characters between Hf and H atoms, we calculate the q_e_/atom from Hf to H by the Bader charge analysis for *I*4/*mmm*, *Cmma* and *P*2_1_/*m* at different pressures, as shown in Figure SI. We can see that about 1.22 electrons and 1.12 electrons transfer from each Hf to H atom for *I*4/*mmm* and *Cmma* structure, at 100 and 200 GPa. For *P*2_1_/*m*, the number of electron transfer from each Hf is 1.29 and 0.42 at 250 GPa, which originates from its two different Hf site. In addition, the pressure dependence of δ (δ donates the values of charge transfer from Hf to H atom) for the above three phases is different. The δ depended on pressure in *Cmma* is smaller than that in *I*4/*mmm*. While turning to *P*2_1_/*m*, it exists two forms due to two inequitable Hf sites. The change of charge transfer may be related to the structural evolution. Overall, the ELF, the difference charge density and Bader analysis results reveal that the ionic bonds were formed between Hf and H, and HfH_2_ was classified as a ionic cyrstal with the charge transferring from Hf to H atom.

We calculated the electron phonon coupling (EPC) parameter λ, the logarithmic average phonon frequency ω_log_, and the electronic DOS at the Fermi level N(E_f_) for *I*4/*mmm*, *Cmma* and *P*2_1_/*m*. The *T*_*c*_ was estimated by using the Allen-Dynes modified McMillan equation^25^ 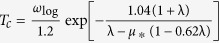,. For materials with λ < 1. 5, this equation has been found to be accurate. The Coulomb parameter set μ^*^ = 0.1–0.13 are adopted for HfH_2_. According to our calculations, the *λ* are 0.33, 0.64 and 0.87 for *I*4/*mmm* (at 1 atm), *Cmma* (at 180 GPa) and *P*2_1_/*m* (at 260 GPa). The estimated *T*_*c*_ of *I*4/*mmm*, *Cmma* and *P*2_1_/*m*, are 47–193 mK at 1 atm, 5.99–8.16 K at 180 GPa and 10.62–12.8 K at 260 GPa, respectively. We note that *I*4/*mmm* has a lower *T*_*c*_ value relative to the other two structures. For the *I*4/*mmm* (at 1 atm), the EPC parameter λ is 0.33, which indicate the electron-phonon interaction is fairly weak. In addition, the electronic DOS at the Fermi level N(E_f_) for the phase *I*4/*mmm* is relatively small (3.978 states/spin/Ry/Unit cell). So the weak electron phonon coupling λ and small N(E_f_) are the main factors, which lead to the low *T*_*c*_. To study the pressure dependence of the superconducting critical temperature *T*_*c*_ of *I*4/*mmm*, *Cmma* and *P*2_1_/*m*, the λ, ω_log_ and N(E_f_) were calculated as summarized in Table 1. With the increasing pressure, the value of ω_log_ is increased in *I*4/*mmm*, while it is diminished in *Cmma* and *P*2_1_/*m*. For N(E_f_) and λ parameters, they both decrease as pressure increased, which mainly lead to the decrease of the *T*_*c*_ values for the above three structures.

## Conclusion

In summary, we have extensively investigated structures and examined the structural stability of HfH_2_ at high pressures up to 300 GPa through *ab initio* evolutionary simulations. Three structures *I*4/*mmm*, *Cmma* and *P*2_1_/*m* are predicted, and all of them are energetically much superior to others phases. The electronic structures are characterized as conductors with band overlap for *I*4/*mmm*, *Cmma* and *P*2_1_/*m* phases. The measured superconducting transition temperature *T*_*c*_ values for *I*4/*mmm*, *Cmma*, and *P*2_1_/*m* are 47–193 mK (at 1 atm), 5.99–8.16 K (at 180 GPa) and 10.62–12.8 K (260 GPa). Further analysis of the bonding nature shows that charges transfer from the hafnium to hydrogen with ionic bonds in HfH_2_. The current study has great implications for researching other transition metal hydrides.

## Methods

We have used the evolutionary algorithm USPEX code (Universal structure predictor: Evolutionary Xtallography)^26^,^27^,^28^ for crystal structure prediction to extensively explore the high-pressure phases of HfH_2_ system at zero temperature. In the evolutionary structural predictions, the first generation of structures was always created randomly and its population size is 20–60 structures, increasing with system size. Every subsequent generation is produced from the best 60% of the previous generation. Moreover, the lowest-enthalpy structures always survived into the next generation. New structures are produced by variation operator heredity (60%), permutation (10%), and lattice mutation (30%). The energetic calculations and electronic structure calculations presented here are performed within density functional theory, carried out within the Vienna ab initio simulation package (VASP)^29^. The generalized gradient approximation with Perdew-Burke-Ernzerh functinal^30^ for the exchange correlation is employed. The projector-augmented wave (PAW)^31^ method is adopted with valence electrons of 5*d*^2^6 *s*^2^ and 1 *s*^1^ and cutoff radii of 2.5 and 0.8 a.u. for Hf and H atoms, respectively. The electronic wave functions were expanded in a plane-wave basis set with a cutoff energy of 800 eV and appropriate Monkhorst-Pack meshes were chosen for all structures to ensure that enthalpy calculations are well converged to better than 1 meV/atom. In the geometrical optimization, all forces on atoms were converged to less than 0.005 eV/Å. We used the Bader charge analysis^32^,^33^,^34^ to calculate the electronic charge transfer. To determine the dynamical stability of the studied structures, the phonon calculations are carried out using a supercell approach^35^ with the PHONOPY code^36^. Electron-phonon coupling (EPC) calculations were carried out using the linear response theory through the Quantum ESPRESSO package^37^. The kinetic energy cutoff was set 90 Ry. And the q-point mesh of the electron-phonon interaction matrix element adopted 4 × 4 × 4, 4 × 4 × 3 and 2 × 3 × 3 for *I*4/*mmm*, *Cmma* and *P*2_1_/*m*, respectively.

## Additional Information

**How to cite this article**: Liu, Y. *et al.* First-principles study on the structural and electronic properties of metallic HfH_2_ under pressure. *Sci. Rep.* **5**, 11381; doi: 10.1038/srep11381 (2015).

## Supplementary Material

Supplementary Information

## Figures and Tables

**Table 1:** The calculated electron-phonon coupling parameters, electronic density of states at the Fermi level N(E _f_) (states/spin/Ry/Unit cell), the logarithmic average phonon frequency ω_log_, and superconducting critical temperatures *T*_*c*_ of *I*4/*mmm*, *Cmma* and *P*_1_/*m* at different pressures.

Structure	P(GPa)	*ω*_log_(K)	N(ε_f_)	*λ*	*T*_*c*_(K)μ* = 0.1	*T*_*c*_(K)μ* = 0.13
*I*4/*mmm*	1 atm	183.819	3.976	0.327	0.192	0.047
	10	201.203	3.486	0.294	0.081	0.012
	30	229.208	2.877	0.26	0.021	0.001
	50	253.173	2.497	0.242	0.008	0
*Cmma*	180	292.708	6.195	0.643	8.159	5.988
	240	285.239	5.191	0.59	6.207	4.322
*P*2_1_/*m*	260	232.059	10.405	0.871	12.804	10.62
	280	218.68	8.322	0.787	7.962	9.911

## References

[b1] Ashcroft N. Metallic Hydrogen: A High-Temperature Superconductor? Phys. Rev. Lett. 21, 1748–1749 (1968).

[b2] Barbee T., García A. & Cohen M. L. First-principles prediction of high-temperature superconductivity in metallic hydrogen. Nature 340, 369–371 (1989).

[b3] Zhang L. *et al.* Ab initio prediction of superconductivity in molecular metallic hydrogen under high pressure. Solid State Commun. 141, 610–614 (2007).

[b4] Loubeyre P., Occelli F. & LeToullec R. Optical studies of solid hydrogen to 320 GPa and evidence for black hydrogen. Nature 416, 613–617 (2002). 11948345 10.1038/416613a

[b5] Duan D. *et al.* Pressure-induced metallization of dense (H_2_S)_2_H_2_ with high-Tc superconductivity. Sci. rep. 4, 6968, (2014). 25382349 10.1038/srep06968 PMC4225546

[b6] Drozdov A. P., Eremets M. I. & Troyan I. A. Conventional superconductivity at 190 K at high pressures *arXiv:1412.0460* (2014). 10.1038/nature14964 26280333

[b7] Duan D. *et al.* Pressure-induced decomposition of solid hydrogen sulfide. *arXiv preprint arXiv*:1501, 01784, (2015).

[b8] Zabel H. *et al.* Hydrogen in thin epitaxial metal films and superlattices: structure, magnetism, and transport. J. Magn. Magn. Mater. (Netherlands) 198, 264–266 (1998).

[b9] Kalita P. E. *et al.* Equation of state of TiH_2_ up to 90 GPa: A synchrotron x-ray diffraction study and ab initio calculations. J. Appl. Phys. 108, 043511, (2010).

[b10] Bowman R., Venturini E., Craft B., Attalla A. & Sullenger D. Electronic structure of zirconium hydride: A proton NMR study. Phys. Rev. B 27, 1474–1488 (1983).

[b11] Sidhu S. S. & McGuire J. C. An X-Ray Diffraction Study of the Hafnium-Hydrogen System. J. Appl. Phys. 23, 1257 (1952).

[b12] Slocombe D. R., Kuznetsov V. L., Grochala W., Williams R. J. & Edwards P. P. Superconductivity in transition metals. *Philosophical transactions*. Series A, Mathematical, physical, and engineering sciences 373, (2015). 10.1098/rsta.2014.0476 25666075

[b13] Züttel A. Materials for hydrogen storage. Mater. Today 6, 24–33 (2003).

[b14] Satterthwaite C. & Toepke I. Superconductivity of Hydrides and Deuterides of Thorium. Phys. Rev. Lett. 25, 741–743 (1970).

[b15] Íñiguez J., Yildirim T., Udovic T., Sulic M. & Jensen C. Structure and hydrogen dynamics of pure and Ti-doped sodium alanate. Phys. Rev. B 70, 060101 (2004).

[b16] Sandrock G., Gross K. & Thomas G. Effect of Ti-catalyst content on the reversible hydrogen storage properties of the sodium alanates. J. Alloy. Compd. 339, 299–308 (2002).

[b17] Yamanaka S., Miyake M. & Katsura M. Study on the hydrogen solubility in zirconium alloys. J. Nucl. Mater. 247, 315–321 (1997).

[b18] Yamanaka S. *et al.* Characteristics of zirconium hydride and deuteride. J. Alloy. Compd. 330, 99–104 (2002).

[b19] Konashi K., Ikeshoji T., Kawazoe Y. & Matsui H. A molecular dynamics study of thermal conductivity of zirconium hydride. J. Alloy. Compd. 356-357, 279–282 (2003).

[b20] Kenji K. *et al.* in Proceedings of the 2006 international congress on advances in nuclear power plants-ICAPP'06 (2006).

[b21] Gao G., Bergara A., Liu G. & Ma Y. Pressure induced phase transitions in TiH2. J. Appl. Phys. 113, 103512 (2013).

[b22] Huang X. *et al.* Structural stability and compressive behavior of ZrH2under hydrostatic pressure and nonhydrostatic pressure. RSC Adv. 4, 46780–46786 (2014).

[b23] Quijano R. & de Coss R. Electronic structure and energetics of the tetragonal distortion for TiH_2_, ZrH_2_, and HfH_2_: A first-principles study. Phys. Rev. B 80, 184103 (2009).

[b24] Born M. & Huang K. Dynamical Theory of Crystal Lattice, Oxford University Press, Oxford (1954).

[b25] Allen P. B. & Dynes R. Transition temperature of strong-coupled superconductors reanalyzed. Phys. Rev. B 12, 905 (1975).

[b26] Oganov A. R. & Glass C. W. Crystal structure prediction using ab initio evolutionary techniques: principles and applications. J. Chem. Phys. 124, 244704 (2006). 16821993 10.1063/1.2210932

[b27] Oganov A. R., Lyakhov A. O. & Valle M. How Evolutionary Crystal Structure Prediction Works–and Why. Acc. Chem. Res. 44, 227–237 (2011). 21361336 10.1021/ar1001318

[b28] Lyakhov A. O., Oganov A. R., Stokes H. T. & Zhu Q. New developments in evolutionary structure prediction algorithm USPEX. Comput. Phys. Commun. 184, 1172–1182 (2013).

[b29] Kresse G. & Furthmüller J. Efficiency of ab-initio total energy calculations for metals and semiconductors using a plane-wave basis set. Comp. Mater. Sci. 6, 15–50 (1996). 10.1103/physrevb.54.11169 9984901

[b30] Perdew J. P., Burke K. & Ernzerhof M. Generalized gradient approximation made simple. Phys. Rev. Lett. 77, 3865 (1996). 10062328 10.1103/PhysRevLett.77.3865

[b31] Kresse G. & Joubert D. From ultrasoft pseudopotentials to the projector augmented-wave method. Phys. Rev. B 59, 1758 (1999).

[b32] Bader R. F. Atoms in molecules. *Accounts Chem*. *Res*. 18, 9–15 (1985).

[b33] Henkelman G., Arnaldsson A. & Jónsson H. A fast and robust algorithm for Bader decomposition of charge density. Comp. Mater. Sci. 36, 354–360 (2006).

[b34] Tang W., Sanville E. & Henkelman G. A grid-based Bader analysis algorithm without lattice bias. J. Phys. Condens. Mat. 21, 084204 (2009). 10.1088/0953-8984/21/8/084204 21817356

[b35] Parlinski K., Li Z. & Kawazoe Y. First-principles determination of the soft mode in cubic ZrO 2. Phys. Rev. Lett. 78, 4063 (1997).

[b36] Togo A., Oba F. & Tanaka I. First-principles calculations of the ferroelastic transition between rutile-type and CaCl_2_-type SiO_2_ at high pressures. Phys. Rev. B 78, 134106 (2008).

[b37] Giannozzi P. *et al.* QUANTUM ESPRESSO: a modular and open-source software project for quantum simulations of materials. J. Phys. Condens. Mat. 21, 395502 (2009). 10.1088/0953-8984/21/39/395502 21832390

