# Supplementary material for: First-principles study on the structural and electronic properties of metallic HfH2 under pressure
Source: Sci Rep. 2015 Jun 22;5:11381. doi: 10.1038/srep11381 (PMC4476111; doi:10.1038/srep11381)

Supplementary information

First-principles study on the structural and electronic properties of metallic HfH_2 under pressure

Yunxian Liu[1], Xiaoli Huang[1], Defang Duan[1], Fubo Tian[1], Hanyu Liu[2], Da Li[1], Zhonglong Zhao[1], Xiaojing Sha[1], Hongyu Yu[1], Huadi Zhang[1], Bingbing Liu[1], and Tian Cui[1,*]

[1]*State Key Laboratory of Superhard Materials, College of Physics, Jilin University, Changchun 130012, People's Republic of China*

[2]*Department of Physics and Engineering Physics, University of Saskatchewan, Saskatoon, Canada, S7N 5E2*

*Corresponding Author: E-mail: cuitian@jlu.edu.cn

Table SI| Structural parameters of our predicted *I*4/*mmm*, *Cmma* and $P2_1/m$ structures for HfH_2 at selected pressure.

Space group Pressure	Lattice parameters (Å, °)	Atomic coordinates (fractional)	Sites
*I*4/*mmm*	a=3.48130	H1 -0.50000 -0.00000 0.25000	4d
1 atm	b=3.48130	Hf1-0.50000 -0.50000 0.50000	2a
	c=4.29830		
	α=β=γ=90		
*P*4/*nmm*	a=2.65040	H1 0.50000 0.50000 0.50000	2b
200 GPa	b=2.65040	H2 1.00000 0.50000 0.80958	2c
	c=4.38460	Hf1 1.00000 0.50000 0.23170	2c
	α=β=γ=90		
Cmma	a=3.77120	H1 -0.50000 -0.00000 0.25000	4a
200 GPa	b=3.71190	H3 1.00000 0.75000 -0.31150	4g
	c=4.39740	Hf1 1.00000 0.75000 -0.73205	4g
	α=β=γ=90		
$P2_1/m$	a=6.77780	H1 0.30099 0.75000 0.62528	2e
300 GPa	b=2.63920	H3 0.70584 0.75000 0.72289	2e
	c=2.81430	H4 0.14140 0.75000 0.23218	2e
	α=90	H6 1.00948 0.25000 0.25034	2e
	β=89.9326	Hf1 0.55556 0.25000 0.87509	2e
	γ=90	Hf3 0.85121 0.75000 0.25169	2e

Table SII| Elastic constants C_{ij}(GPa) of *I*4/*mmm*, *P*4/*nmm, Cmma* and *P*2$_1$/*m* at 100, 180, 180 and 250 GPa, respectively.

*I*4/*mmm*	C_{11}	C_{33}	C_{44}	C_{66}	C_{12}	C_{13}	
	7161.3	4024.19	845.70	498.43	2456.79	2998.98	
Cmma	C_{11}	C_{22}	C_{33}	C_{44}	C_{55}	C_{66}	C_{12}
	7243.01	7242.8	7897.25	478.47	961.45	1687.76	6127.59
	C_{13}	C_{23}					
	5907.05	4799.58					
*P*2$_1$/*m*	C_{11}	C_{22}	C_{33}	C_{44}	C_{55}	C_{66}	C_{12}
	8813.52	7701.95	8369.76	1329.87	1183.25	1164.84	4596.36
	C_{13}	C_{15}	C_{23}	C_{25}	C_{35}	C_{46}	
	6916.22	-937.4	5037.86	-455.06	-421.98	53.94	
*P*4/*nmm*	C_{11}	C_{33}	C_{44}	C_{66}	C_{12}	C_{13}	
	7225.13	7307.83	366.49	-561.72	5721.53	5383.86	

Figure SI | Bader analysis for the decreasing charge value of Hf in *I*4/*mmm*, *Cmma* and $P2_1/m$ structures at different pressures. δ donates the values of charge transfer from Hf to a H atom.

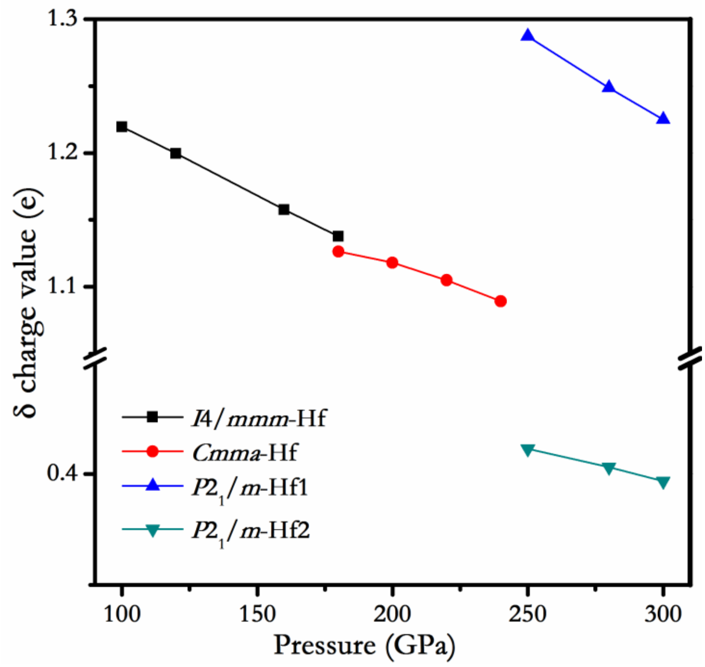

Supplement: Supplementary Information [file srep11381-s1.pdf]
